# Medical Extracts: Practical and Philosophical—No. 1

**Published:** 1873-04

**Authors:** L. B. Anderson

**Affiliations:** Virginia


### MEDICAL EXTRACTS: PRACTICAL AND PHILOSOPHICAL—NO. I.

### *FATAL VACCINATION.*

BY L. B. ANDERSON, M.D., OF VIRGINIA.

At a country store in the county of Hanover, Virginia, a goodly number of persons assembled on Saturday, the 15th of February, 1873, to be vaccinated by a country merchant. Several submitted to the operation, only two of whom were impressed by the matter used. One of these was Mr. H., a gentleman fifty years of age, somewhat addicted to the immoderate use of spirituous drinks, but otherwise of good health and constitution. The virus was taken directly from the arm of a child some nine days after vaccination. The first crust had been forcibly removed before its maturity, and another had formed, under the edges of which there was a free discharge of pus. A puncture was made with the point of a lancet at the lower line of the upper third of the arm, the scab raised from the arm of the child, and the "matter" secured and introduced in the puncture. Mr. H. felt no uneasiness, or any effect, from the operation until the next morning, when, on waking, he complained of headache, nausea and chilliness. He ate something and soon vomited freely. In the meantime the arm began to inflame and swell, both passing towards the hand. The pain was so intense during Sunday night he was unable to sleep. The pain, swelling and redness rapidly increased, accompanied with a burning fever during Monday. Learning on Tuesday that "lead-water" was being used in a similar case, he applied it freely during the forenoon, with no relief of pain, however, or abatement of the symptoms. Dr. F. was summoned to see him, but being too unwell to visit him, he directed applications of "warm water and vinegar" to the arm. This was continued, with no abatement of the symptoms, until Dr. S. directed the arm to be painted with tr. iodine, a large dose of calomel to be taken, and twenty drops mur. tr. iron to be given three times a day. The iodine was applied freely, soon after which the writer saw him.

At two o'clock P.M. Thursday, the fifth day after the operation was performed, the pulse was frequent, but neither feeble or strong; the flesh was warm, but not very hot; the countenance was expressive of anxiety; the stomach and bowels quiet; the kidneys acting well; the breathing nearly natural; the tongue a little coated; the left arm very much swollen, abraded and painful; the hand and fingers also greatly tumefied and painful. There was a free discharge of a yellowish, oily, acrid fluid from the abrasions. The appearance was very unhealthy in its whole extent. Even the abraded spots, comprising one-fourth of the surface, where no iodine was applied, presented a livid, indolent, shreddy, spongy appearance.

Chlorate of potass. in five-grain doses every two or three hours—thickened milk poultices, covered with camphorated oil and laudanum, to be applied every four hours to the arm—constituted the treatment directed. He remained quiet under this treatment until Friday morning, when he became occasionally delirious. At twelve o'clock he was bathed in a profuse perspiration. At nine o'clock P.M. he expired. I saw him only once, and can give no description of the changes which occurred on the sixth and last day. I infer that the absorption of morbific matters into the circulation, and their depressing effects upon the nervous centres, and disorganizing influence on the blood, produced death.

Mr. A., thirty years of age, spare, delicate, and of a nervous temperament, was "*vaccinated*" on the same day, by the same person and with the same matter that Mr. H. was. He was taken in the same way on the following morning, and at ten o'clock A.M. had a chill which lasted nearly two hours, followed by a very active fever, and extensive and painful inflammation of the arm. Ten grains of calomel were given that night, and a saline aperient the next morning, which operated well; after which, a thickened milk poultice, saturated with the water of the acetate of lead, was applied to the whole arm, and chlorate of potass. in five-grain doses was given every three hours internally. Diet to be buttermilk chiefly, and if the fever passed off, to give sixteen grains quinine during the following day. On Tuesday, the inflammation had subsided near the puncture, but increased considerably below, near and below the elbow. On Wednesday, he was much better, the fever having passed off, and the inflammation having become more circumscribed. On the evenings of Thursday and Friday he had a slight rise of fever. Quinine was given him on Saturday, and the other agents recommended were to be continued. On Saturday, the inflammation was confined almost entirely to the elbow and the parts immediately below. The former treatment was continued, with the addition of sixteen grains quinine to be given in forenoon. There was less tumefaction, redness and pain in the arm, though the pulse was unsteady, the countenance indicating excitement, and there were flying neuralgic pains about the head and face, with considerable nervous impressibility. The quinine, chlo. potass., lead-water and poultices were continued, with an occasional aperient of castor oil and spirits turpentine, mild and nourishing diet, until the patient was relieved.

---

RINGWORM—CITRINE OINTMENT.—In the application of the pure, fresh citrine ointment (ung hydr. nit) seldom fails to cure this disease. Apply it twice a day, and keep the part clean.

